# The rising burden of diabetes and state-wise variations in India: insights from the Global Burden of Disease Study 1990–2021 and projections to 2031

**DOI:** 10.3389/fendo.2025.1505143

**Published:** 2025-05-12

**Authors:** Shubham Chauhan, Mahalaqua Nazli Khatib, Suhas Ballal, Pooja Bansal, Kiran Bhopte, Abhay M. Gaidhane, Balvir S. Tomar, Ayash Ashraf, M. Ravi Kumar, Ashish Singh Chauhan, Muhammed Shabil, Diptismita Jena, Ganesh Bushi, Prakasini Satapathy, Lara Jain, Vaibhav Jaiswal, Manvi Pant

**Affiliations:** ^1^ Evidence for Policy and Learning, Global Center for Evidence Synthesis, Chandigarh, India; ^2^ Division of Evidence Synthesis, Global Consortium of Public Health and Research, Datta Meghe Institute of Higher Education, Wardha, India; ^3^ Department of Chemistry and Biochemistry, School of Sciences, JAIN (Deemed to be University), Bangalore, Karnataka, India; ^4^ Department of Allied Healthcare and Sciences, Vivekananda Global University, Jaipur, Rajasthan, India; ^5^ Infotech Education Society (IES) Institute of Pharmacy, IES University, Bhopal, Madhya Pradesh, India; ^6^ Jawaharlal Nehru Medical College, and Global Health Academy, School of Epidemiology and Public Health, Datta Meghe Institute of Higher Education, Wardha, India; ^7^ Institute of Pediatric Gastroenterology and Hepatology, National Institute of Medical Sciences University, Jaipur, India; ^8^ Chandigarh Pharmacy College, Chandigarh Group of College, Mohali, Punjab, India; ^9^ Department of Chemistry, Raghu Engineering College, Visakhapatnam, Andhra Pradesh, India; ^10^ Division of Research and Innovation, Uttaranchal Institute of Pharmaceutical Sciences, Uttaranchal University, Dehradun, India; ^11^ University Center for Research and Development, Chandigarh University, Mohali, Punjab, India; ^12^ Department of Computer Science and Engineering, Graphic Era (Deemed to be University), Dehradun, India; ^13^ School of Pharmaceutical Sciences, Lovely Professional University, Phagwara, India; ^14^ Medical Laboratories Techniques Department, AL-Mustaqbal University, Hillah, Babil, Iraq; ^15^ Department of Dentistry, Graphic Era (Deemed to be University), Dehradun, India; ^16^ New Delhi Institute of Management, Delhi, India

**Keywords:** diabetes, incidence, mortality, DALYs, India, GBD study, ARIMA, regional disparities

## Abstract

**Background:**

Diabetes is a major public health concern in India, contributing significantly to morbidity and mortality. With variations in disease burden across states, a detailed understanding of trends in incidence, prevalence, and Disability Adjusted Life Years (DALYs) is essential for targeted interventions.

**Methods:**

This study utilized Global Burden of Disease (GBD) data from 1990 to 2021 to examine trends in diabetes across Indian states. Age-standardized incidence, prevalence, mortality, and DALYs were analyzed using Join point regression to estimate Annual Percentage Change (APC). Autoregressive Integrated Moving Average (ARIMA) models were employed to project diabetes trends up to 2031.While the GBD data provide robust national and regional estimates, their modeled nature may not capture the full spectrum of local epidemiological variations.

**Results:**

Diabetes incidence increased from 162.74 to 264.53 per 100,000 between 1990 and 2021, with an APC of 0.63%. Joinpoint analysis identified episodic surges in incidence, with APCs of 2.25% during 1996–1999 and 2.07% during 2005–2011, suggesting intervals of accelerated increase relative to the gradual progression typically observed in chronic conditions. Mortality rose from 23.09 to 31.12 per 100,000 (APC: 0.12%). Southern and Western states, such as Tamil Nadu and Goa, exhibited the highest prevalence and DALYs. Forecasted trends indicate that by 2031, the prevalence will reach 8585.45 per 100,000, and DALYs will exceed 1241.57 per 100,000.

**Conclusion:**

The burden of diabetes in India has risen markedly over the past three decades. These findings underscore the urgent need for health policies that emphasize lifestyle modifications and improved healthcare access. A comprehensive approach that integrates primary prevention through community-based health education, dietary counseling, and initiatives to promote physical activity with secondary prevention measures such as systematic screening and timely clinical management, is essential for effective diabetes control and management in high-burden states.

## Introduction

Diabetes is a serious, chronic condition characterized by high blood glucose levels, which arise due to disturbances in β-cell biology impacting insulin function (1, 2). Diabetes mellitus is a widespread medical condition that has the potential to cause significant harm and has become more common over the past few decades, making it a major public health issue of the twenty-first century (3). Globally, diabetes is a leading cause of morbidity and mortality, significantly contributing to the overall disease burden (4). In 2021, approximately 529 million people were affected by diabetes (5). Diabetes affects people across all age groups, genders, and regions, ranking among the leading causes of death and illness worldwide (6). Diabetes is also a significant risk factor for ischaemic heart disease and stroke, which the Global Burden of Disease (GBD) identified as the primary and secondary leading causes of the global disease burden, respectively (5). Over the past 25 years, the global burden of diabetes has progressively increased, with India accounting for a substantial share of this worldwide trend. The United Nations classifies diabetes among the priority non-communicable diseases, emphasizing the critical role of national governments in formulating comprehensive, multisectoral strategies for NCD prevention and control (7). In India, diabetes has become a pressing concern, given its rapid rise in both rural and urban areas (8, 9). Higher concurrent prevalence of diabetes mellitus (DM) and hypertension (HTN) was observed in older individuals, those with a high BMI, those with a waist-to-hip ratio greater than 1, as well as individuals with a higher wealth index and higher education levels.

NCDs account for approximately 60% of all deaths in India, with diabetes, hypertension, and obesity being particularly prevalent (10, 11). Key behavioral risk factors include physical inactivity, unhealthy diets, tobacco use, and harmful alcohol consumption (12). According to the National Family Health Survey (NFHS-5, 2019-2021), the overall prevalence of diabetes among adults in India is estimated to be 6.5% (13). Another study using data from the same survey reported a higher prevalence of 16.1% when including both diagnosed and undiagnosed cases (14). There are 17 Sustainable Development Goals, with Goal 3 being explicitly focused on health, aiming to “ Ensure healthy lives and promote well-being for all at all ages”. Each goal encompasses specific targets, and Target 3.4 addresses NCDs. Its objective is to “reduce premature mortality from NCDs by one third through prevention and treatment, while also promoting mental health and well-being” by the year 2030 (15, 16). In 2021, an estimated 101 million individuals had diabetes (10). This positions the country as a significant epicenter for diabetes related health issues. However, the national level analysis often conceals critical regional disparities, making it essential to have detailed, state specific data on diabetes and risk factors to guide targeted policy and interventions. A comprehensive 30-year analysis (1990–2021) using the latest GBD 2021 data is needed to provide insights into these variations, as existing research has largely focused on national trends without accounting for state-wise differences, which are crucial for understanding localized public health challenges. To effectively address the NCD burden, comprehensive country level data are needed to monitor epidemiological trends and inform policy. This study aims to fill these gaps by providing a comprehensive analysis of state-wise trends in diabetes incidence, prevalence, mortality, and Disability-Adjusted Life Years (DALYs) from 1990 to 2021. The objective of this research is to analyze these long-term trends to offer actionable insights that can guide the development of state-specific diabetes prevention and management strategies. This study aims to support policymakers in understanding regional variations, identifying high-risk areas, and formulating targeted interventions that can address the escalating diabetes crisis in a more nuanced and effective manner. In doing so, it contributes to the broader goal of optimizing diabetes control strategies in India by providing evidence-based recommendations that consider both the geographic and demographic diversity of the country’s diabetes burden.

## Methods

### Data sources

We conducted an analysis of epidemiological data provided by the GBD study, which is maintained by the Institute for Health Metrics and Evaluation at the University of Washington, Seattle (17).We analyzed age-standardized metrics including incidence, prevalence, mortality, and DALYs for diabetes across various states in India. For this analysis, we selected the aggregate measure encompassing both type-1 diabetes (T1D) and type-2 diabetes (T2D) to capture the overall burden of diabetes mellitus.

### Statistical analysis

#### Join point regression analysis

We applied join point regression analysis to identify points where significant changes in the trend of diabetes metrics occurred over time. This method allowed for the detection and estimation of Annual Percentage Changes (APC) in incidence and mortality rates. It models the data using changes in slope determined by estimated breakpoints. The model can be represented as: y(t)= β_0_ ​+ β_1_​t + ∑^kj =1^ ​β_j+1_ ​(t−τ_j_​)+​, where (t−τ_j_ ​)_+​_ is zero for t ≤τ_j_ and (t- τ_j_​) for t > τ _j_. Each τ_j_​ represents a “joinpoint” indicating a potential change in the slope of the linear segments, and continuity constraints are applied at each joinpoint to ensure a smooth fit. The number and location of these joinpoints are determined through permutation tests and the U.S. National Cancer Institute’s Joinpoint Regression Program (Version 5.2.0). By statistically identifying these points of change, joinpoint regression provides a robust means of pinpointing when significant shifts in diabetes incidence and mortality occurred within the study period (18).

### Forecasting (ARIMA) model

For the projection of diabetes age standardized prevalence and DALYs up to 2031, we utilized Autoregressive Integrated Moving Average (ARIMA) models. These models were selected for their ability to handle non-stationary time series data by incorporating terms for auto regression, differencing, and moving averages. R (4.4.1 version) was used to generate estimation with the best model of automatically selected P, D and Q (19). In our analysis, we utilized Akaike’s Information Criterion (AIC) and the Bayesian Information Criterion (BIC) to identify the optimal ARIMA model for forecasting. The AIC is defined as: AIC=−2log(L)+2(p+q+k+1) where log(L) represents the log likelihood of the model. The BIC extends the AIC by incorporating a penalization factor that more heavily penalizes models with greater complexity, calculated as: BIC=AIC+[log(T)−2] (p+q+k+1). By minimizing both the AIC and BIC, we ensured that the selected model was robust, effectively capturing the essential dynamics of the data while avoiding overfitting. We selected the model with least AIC and BIC values enabling us to balance the trade-off between model complexity and fit. This methodology supports the reliability and accuracy of our forecasting results. The detailed methodology given elsewhere (20, 21). Choropleth maps were generated using QGIS 3.38.0 ‘Grenoble’, depicting the age-standardized rates for DALYs and APC across different states. Data visualization and subsequent analysis were conducted using Microsoft Excel 2021.

## Results

The analysis of diabetes trends in India between 1990 and 2021 highlights a substantial rise in both the incidence and mortality rates across the country, with significant regional variations. Nationally, the incidence of diabetes increased from 162.74 per 100,000 in 1990 to 264.53 per 100,000 in 2021, with an APC of 0.63% (**Table 1**). Mortality also rose from 23.09 to 31.12 per 100,000, though at a slower rate, with an APC of 0.12%. Tamil Nadu experienced the highest incidence rate in 2021 at 392.41 per 100,000 (APC 0.78%), and Goa had the steepest increase in incidence (APC 0.87%). In contrast, Kerala and Bihar saw more moderate growth in incidence, with APCs of 0.32% and 0.51%, respectively. Mortality trends varied more significantly between states. Uttar Pradesh exhibited the highest rise in mortality (APC 0.73%), while Jharkhand experienced a slight decline in mortality rates (APC -0.21%). Despite having one of the highest incidence increases, Chhattisgarh showed stable mortality rates (APC -0.06%). In contrast, Uttarakhand and Punjab saw substantial increases in both incidence and mortality rates, with Uttarakhand showing one of the highest APCs in mortality (0.46%). Southern and Western India (notably Tamil Nadu, Goa, and Karnataka) bearing a higher overall burden of diabetes compared to Northern and Northeastern regions (Uttar Pradesh and Assam), where growth was slower but steady.

**Table 1:** State-wise trends in age-standardized Incidence and Mortality Rates (per 100,000) for diabetes in India (1990-2019), with annual percentage change (APC).

	Incidence per 100,000 (95% UI)			Mortality per 100,000 (95% UI)		
Country and States	1990	2021	APC	1990	2021	APC
India	162.74 (147.77 to 179.2)	264.53 (240.77 to 289.61)	0.63 (0.57 to 0.68)	23.09 (19.71 to 26.14)	31.12 (27.57 to 34.82)	0.12 (-0.2 to 0.61)
Andhra Pradesh	161.49 (146.45 to 178.69)	252.78 (226.61 to 278.43)	0.57 (0.48 to 0.65)	22.45 (16.74 to 28.4)	25.16 (19.79 to 31.33)	0.27 (-0.08 to 0.76)
Arunachal Pradesh	159.07 (144.24 to 174.97)	256.91 (232.47 to 282.37)	0.62 (0.54 to 0.7)	27.11 (21.33 to 34.31)	34.4 (27.01 to 42.72)	0.26 (-0.04 to 0.69)
Assam	167.7 (151.81 to 186.27)	263.55 (238.12 to 289.76)	0.57 (0.49 to 0.66)	27.28 (21.86 to 32.97)	34.32 (28.9 to 40.64)	0.17 (-0.13 to 0.52)
Bihar	149.61 (134.48 to 166.49)	225.72 (202.18 to 249.72)	0.51 (0.44 to 0.58)	22.77 (18.33 to 27.49)	26.65 (21.75 to 31.81)	0.5 (0.14 to 1.01)
Chhattisgarh	155.77 (140.84 to 172.02)	283.93 (257.8 to 313)	0.82 (0.75 to 0.91)	29.31 (23.17 to 35.46)	43.93 (36.61 to 52.76)	-0.06 (-0.27 to 0.24)
Delhi	168.34 (152.87 to 184.03)	278.87 (251.9 to 306.43)	0.66 (0.57 to 0.74)	29.74 (24.66 to 35.39)	27.94 (23.02 to 33.58)	0.5 (0.03 to 1.09)
Goa	168.08 (153.16 to 183.82)	314.3 (289.17 to 344.61)	0.87 (0.78 to 0.96)	26.61 (20.81 to 32.89)	39.94 (31.24 to 50.07)	0.61 (0.28 to 1.04)
Gujarat	156.61 (141.74 to 173.67)	261.69 (238.21 to 287.54)	0.67 (0.6 to 0.74)	19.65 (16.59 to 22.5)	31.62 (26.65 to 36.53)	0.55 (0.23 to 1.05)
Haryana	143.19 (130.53 to 157.96)	261.29 (236.74 to 289.31)	0.82 (0.75 to 0.92)	17.33 (14.37 to 20.22)	26.85 (22.63 to 31.69)	0.17 (-0.1 to 0.54)
Himachal Pradesh	137.31 (123.44 to 152.09)	234.25 (212.05 to 257.86)	0.71 (0.63 to 0.8)	15.61 (12.81 to 18.97)	18.25 (15.05 to 21.83)	0.35 (0.1 to 0.61)
Jammu & Kashmir and Ladakh	134.06 (121.15 to 147.98)	240.44 (218.44 to 263.03)	0.79 (0.71 to 0.88)	15.25 (11.62 to 19.54)	19.68 (16.26 to 23.02)	0.29 (-0.02 to 0.77)
Jharkhand	170.27 (153.03 to 189.18)	234.42 (209.62 to 261.29)	0.38 (0.31 to 0.44)	27.7 (20.76 to 34.17)	21.87 (18.2 to 26.29)	-0.21 (-0.38 to 0.03)
Karnataka	182.92 (166.33 to 201.3)	306.99 (280.49 to 338.56)	0.68 (0.61 to 0.75)	33.11 (26.59 to 40.52)	46.67 (39.53 to 54.33)	0.41 (0.05 to 0.79)
Kerala	215.09 (195.22 to 235.63)	284.92 (260.96 to 312.14)	0.32 (0.26 to 0.38)	24.95 (18.76 to 30.61)	30.05 (25.57 to 34.86)	0.2 (-0.1 to 0.63)
Madhya Pradesh	146.87 (132.95 to 161.48)	251.55 (225.9 to 275.05)	0.71 (0.63 to 0.79)	18.39 (14.69 to 22.36)	25.57 (21.78 to 29.54)	0.39 (0.1 to 0.81)
Maharashtra	167.9 (152.34 to 184.32)	248.76 (225.82 to 274.34)	0.48 (0.42 to 0.55)	21.34 (17.53 to 24.85)	27.23 (23.21 to 31.93)	0.28 (0.03 to 0.59)
Manipur	183.85 (167.15 to 202.78)	295.27 (267.89 to 324.45)	0.61 (0.53 to 0.68)	37.36 (28.39 to 46.1)	49.54 (39.51 to 61.83)	0.33 (-0.05 to 0.88)
Meghalaya	143.59 (130.16 to 158.83)	227.28 (205.7 to 251.61)	0.58 (0.52 to 0.66)	19.61 (14.97 to 24.58)	28.18 (22.98 to 33.94)	0.44 (0.04 to 0.96)
Mizoram	157.1 (143.5 to 172.67)	234.95 (213.36 to 257.06)	0.5 (0.43 to 0.57)	23.73 (18.65 to 29.55)	25.8 (20.18 to 32.07)	0.09 (-0.22 to 0.52)
Nagaland	143.84 (129.84 to 158.79)	248.97 (224.47 to 273.17)	0.73 (0.65 to 0.81)	17.19 (13.33 to 21.01)	23.73 (18.41 to 29.8)	0.38 (0.01 to 0.93)
Odisha	148.67 (134.72 to 163.28)	246.51 (223.52 to 269.41)	0.66 (0.58 to 0.74)	19.45 (15.41 to 23.44)	26.64 (22.08 to 32.17)	0.37 (0.04 to 0.79)
Other Union Territories	198.42 (181.43 to 218.2)	296.95 (269.67 to 325.97)	0.5 (0.42 to 0.57)	24.28 (19 to 30.38)	31.96 (25.39 to 39.02)	0.32 (-0.06 to 0.81)
Punjab	194.38 (176.75 to 213.12)	302.76 (278.15 to 333.24)	0.56 (0.49 to 0.62)	31.83 (26.85 to 37.47)	45.23 (37.8 to 52.61)	0.42 (0.12 to 0.82)
Rajasthan	126.29 (113.19 to 139.85)	221.07 (200.04 to 244.39)	0.75 (0.67 to 0.85)	10.75 (8.64 to 13.11)	15.25 (12.78 to 18.26)	0.42 (0.09 to 0.82)
Sikkim	149.87 (135.73 to 164.91)	263.94 (239.6 to 289.99)	0.76 (0.68 to 0.85)	22.61 (17.57 to 28.26)	28.43 (22.33 to 36.18)	0.26 (-0.1 to 0.76)
Tamil Nadu	220.54 (200.23 to 243)	392.41 (358.88 to 427.37)	0.78 (0.71 to 0.86)	45.12 (36.02 to 53.51)	64.54 (53.22 to 75.29)	0.43 (0.05 to 0.86)
Telangana	175.87 (160.07 to 193.78)	268.48 (242.57 to 296.32)	0.53 (0.46 to 0.59)	28.6 (21.6 to 36.36)	40.33 (31.61 to 49.51)	0.41 (-0.01 to 0.98)
Tripura	155.86 (141.33 to 171.79)	240.53 (217.92 to 263.96)	0.54 (0.47 to 0.61)	17.03 (13.52 to 21.23)	21.61 (17.32 to 26.5)	0.27 (-0.05 to 0.7)
Uttar Pradesh	145.92 (131.72 to 161.85)	259.69 (233.58 to 286.34)	0.78 (0.69 to 0.87)	16.36 (13.23 to 19.17)	28.28 (23.89 to 33.57)	0.73 (0.36 to 1.19)
Uttarakhand	165.3 (150.61 to 182.41)	300.29 (271.35 to 331)	0.82 (0.74 to 0.9)	33.27 (27.67 to 39.63)	48.71 (40.38 to 57.41)	0.46 (0.11 to 0.91)
West Bengal	140.78 (127.27 to 156.02)	223.14 (200.07 to 247.21)	0.59 (0.51 to 0.66)	17.16 (14.07 to 20.14)	19.72 (16.69 to 22.89)	0.15 (-0.09 to 0.47)

**Figure 1:**
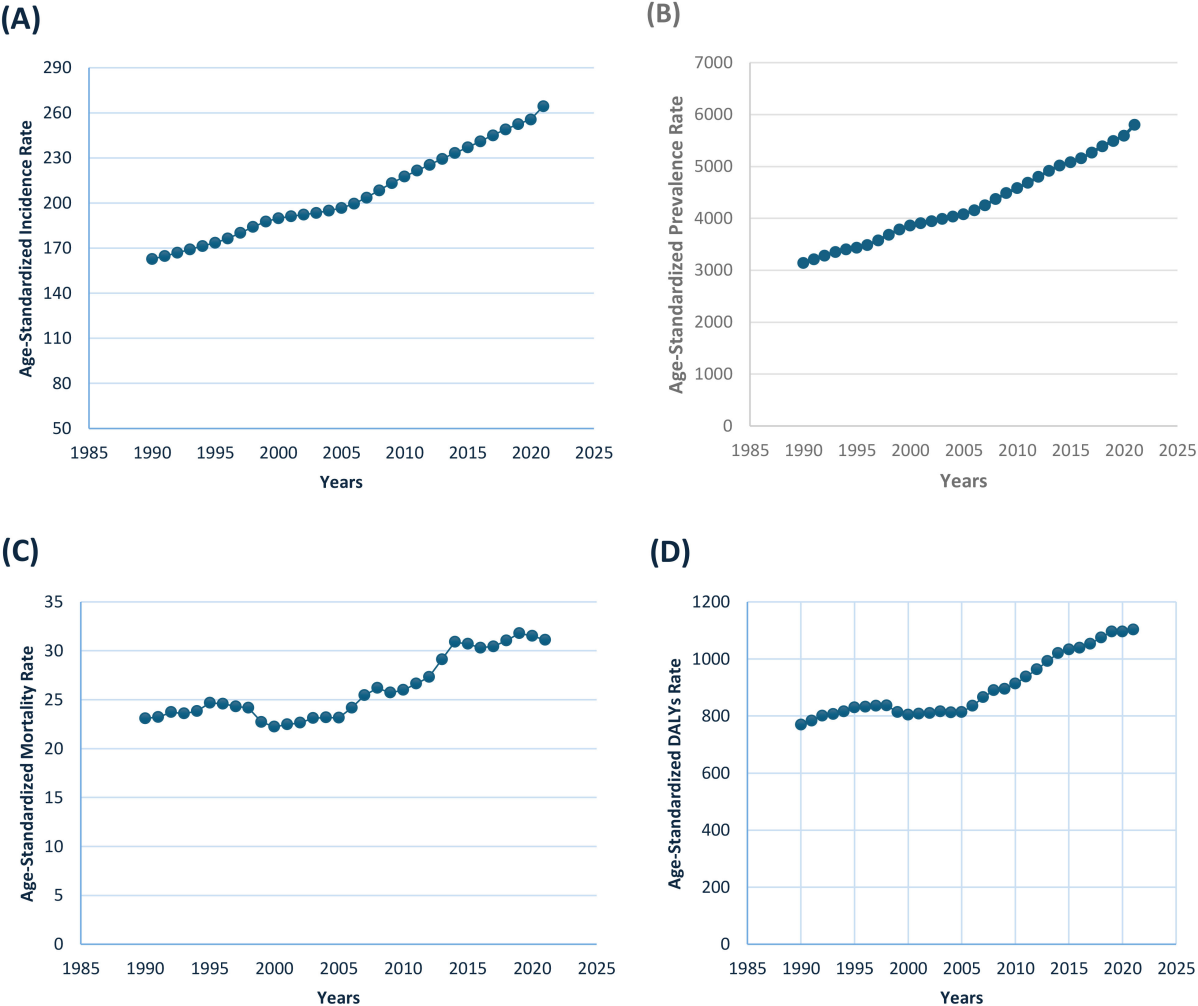
Trends in Age-standardized Rates (per 100,000) of Diabetes in India from 1990 to 2019, showing **(A)** Incidence, **(B)** Prevalence, **(C)** Mortality, and **(D)** Disability-Adjusted Life Years (DALYs).

The analysis of diabetes trends in India from 1990 to 2021 shows a significant and continuous increase in the age-standardized incidence rate (ASIR), prevalence rate (ASPR), mortality rate (ASMR), and DALY. The ASIR rose from 162.74 per 100,000 in 1990 to 264.53 per 100,000 in 2021, with notable growth periods between 1996–1999 and 2015-2021 (**Figure 1**). Similarly, the ASPR nearly doubled during the same period, increasing from 3141.03 per 100,000 in 1990 to 5804.18 per 100,000 in 2021, with significant rises between 2000–2008 and 2015-2021. The ASMR, which began at 23.09 per 100,000 in 1990, saw fluctuations but ultimately rose to 31.12 per 100,000 by 2021, peaking in 2019 (31.8). The DALY burden followed a similar trend, increasing from 769.3 per 100,000 in 1990 to 1102.82 per 100,000 in 2021, with a particularly sharp rise after 2005, reaching over 1000 per 100,000 by 2014.

**Figure 2:**
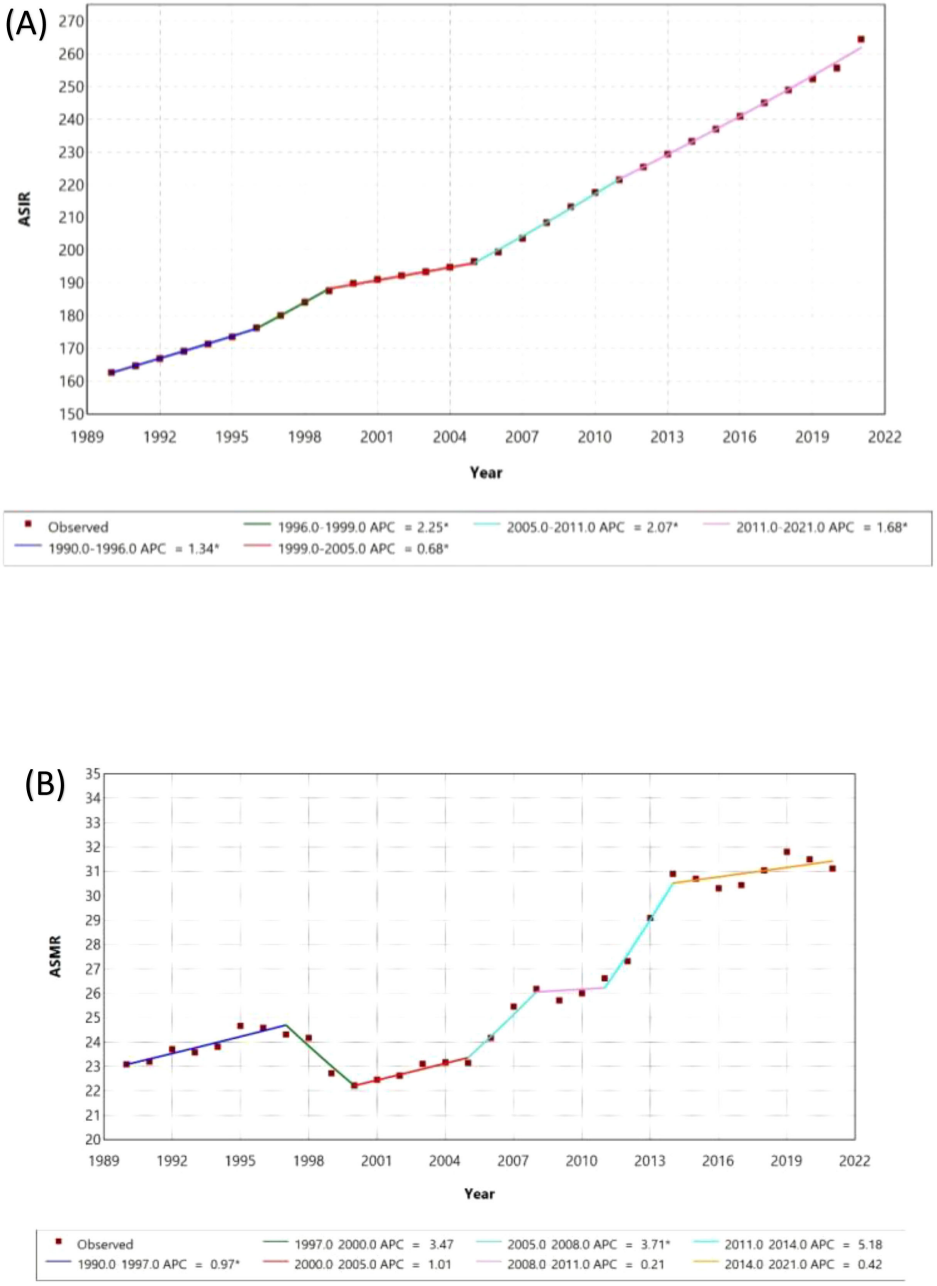
Join point Regression Analysis of Age-Standardized Incidence (ASIR) and Mortality Rates (ASMR) with Annual Percentage Change (APC) from 1990 to 2021. **(A)** ASIR **(B)** ASMR.

Join point analysis from 1990 to 2021 shows a significant rise in the age-standardized incidence rate (ASIR) of diabetes, with an overall average annual percentage change (AAPC) of 1.55% (95% CI: 1.53 to 1.57) (**Supplementary Table 1**). Between 1990 and 1996, the ASIR increased at a modest rate of 1.34% (95% CI: 1.12 to 1.44), followed by a sharp rise from 1996 to 1999, where the APC reached 2.25% (95% CI: 1.83 to 2.45). From 1999 to 2005, the rate of increase slowed to 0.68% (95% CI: 0.49 to 0.80) (**Figure 2**). However, another surge occurred between 2005 and 2011, with an APC of 2.07% (95% CI: 1.93 to 2.37), before slowing again from 2011 to 2021, with an APC of 1.68% (95% CI: 1.57 to 1.74). In contrast, the age-standardized mortality rate (ASMR) exhibited more variability over the study period. The overall AAPC for ASMR was -1.00% (95% CI: 0.91 to 1.08), indicating a general decline in mortality. From 1990 to 1997, mortality increased with an APC of 0.97% (95% CI: 0.40 to 1.58), followed by a sharp decline between 1997 and 2000, with an APC of -3.47% (95% CI: -4.11 to 1.18). Mortality stabilized slightly from 2000 to 2005 (APC 1.01%, 95% CI: -2.81 to 2.25), before rising sharply from 2005 to 2008, with an APC of 3.71% (95% CI: 0.99 to 4.59). This was followed by relative stabilization from 2008 to 2011 (APC 0.21%, 95% CI: -0.77 to 4.68), and a slight decline from 2014 to 2021, with an APC of 0.42% (95% CI: -0.15 to 1.09) (**Figure 2**, **Supplementary Table 1**).

**Figure 3:**
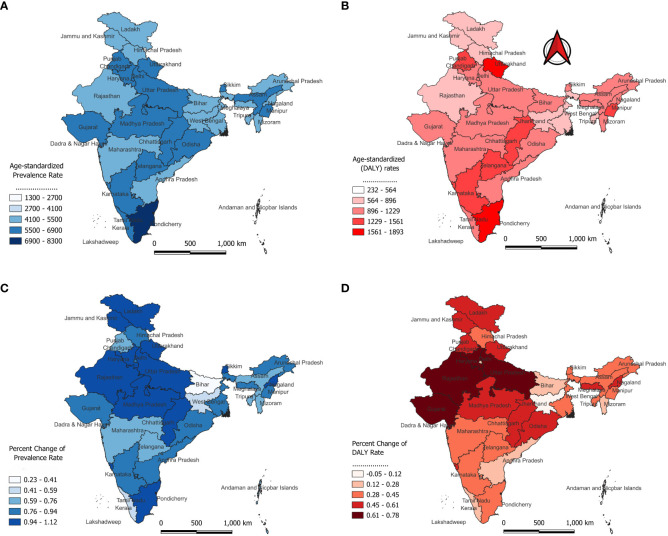
Age-standardized Prevalence and DALYs of Diabetes Across Indian States in 2021 and its Percentage Change from 1990 to 2021; DALY, disability-adjusted life-year. **(A)** Age-standardized Prevalence Rate **(B)** Age-standardized (DALY) Rate **(C)** Percent Change of Prevalence Rate **(D)** Percent Change of DALY Rate.

Tamil Nadu has the highest ASPR at 8,299.55 per 100,000, followed by Goa (6,675.33) and Karnataka (6,663.54) (**Figure 3A**). States like Punjab, Haryana, Uttar Pradesh, and Chhattisgarh also report high prevalence, while Rajasthan, West Bengal, and Nagaland show comparatively lower rates, though still substantial. In terms of DALY rates, Tamil Nadu again leads with 1,893.11 per 100,000 (**Figure 3B**). Other states with high DALY rates include Uttarakhand, Punjab, and Karnataka, whereas Himachal Pradesh, West Bengal, and Nagaland report lower DALY rates. Between 1990 and 2021, Goa saw the highest increase in prevalence (1.12%), followed by Chhattisgarh and Haryana (both 1.09%) (**Figure 3C**). States like Uttar Pradesh, Ladakh, and Jammu and Kashmir also reported notable increases. On the other hand, Bihar, Chandigarh, and Pondicherry had the smallest increases in prevalence at 0.23%. Regarding DALY rates, Gujarat experienced the largest rise (0.68%), while Jharkhand was the only state to show a slight decrease (-0.05) in diabetes burden (**Figure 3D**).

**Figure 4:**
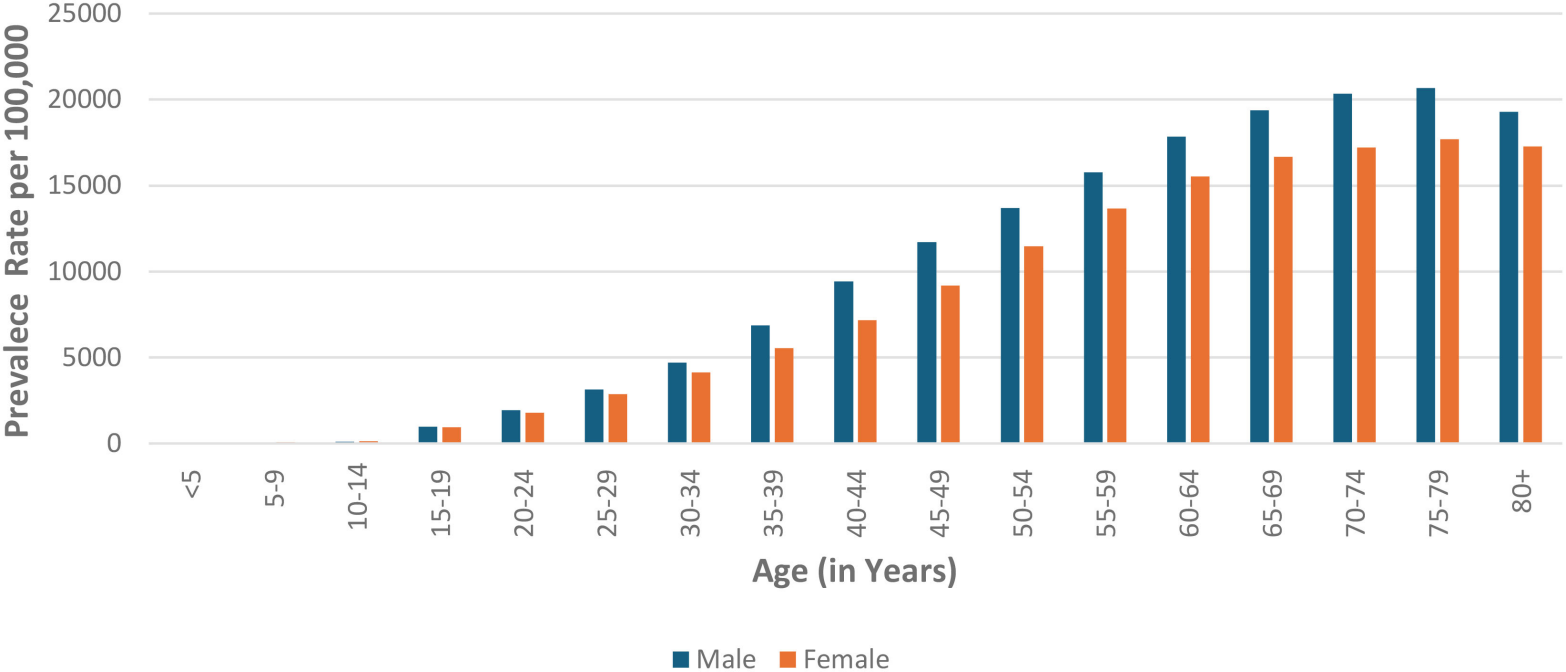
Age-specific prevalence of disease by gender in 2021: Comparison of males and females across different age groups.

The prevalence of diabetes demonstrates a marked increase with advancing age in both males and females, with discernible gender differences across various age cohorts. Starting from a relatively low prevalence in children under the age of five approximately 11.98 per 100,000 in males and 15.11 per 100,000 in females there is a significant rise as individual progress through adolescence (**Figure 4**). Notably, males between the ages of 15 and 19 exhibit a prevalence rate of 981.26 per 100,000, compared to 952.47 per 100,000 in females within the same age group. This gender disparity becomes more pronounced during middle age, culminating in the 70–74 age group, where males experience a peak prevalence of 20,338.99 per 100,000, in contrast to females, who display a prevalence of 17,203.71 per 100,000. While there is a modest decline in diabetes prevalence beyond the age of 80, males consistently exhibit higher rates than females across nearly all age categories.

**Figure 5:**
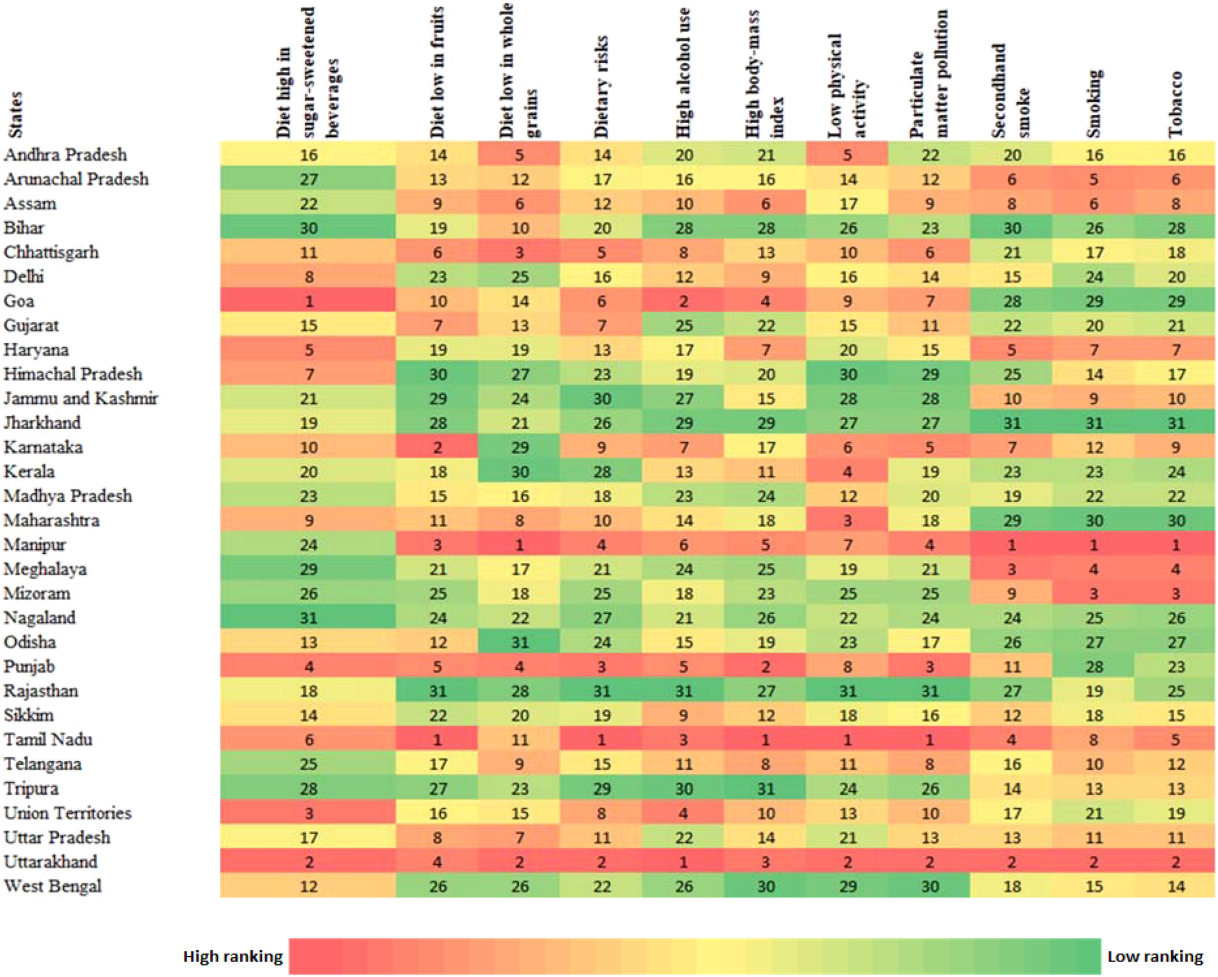
Heat map showing ranking of health risk by state for different types of risk factors of Diabetes in India.

The analysis of health risk factors related to diabetes across Indian states, based on ranking, reveals distinct variations. Goa (ranked 1st) and Tamil Nadu (ranked 1st) show high risks across several categories such as diet high in sugar-sweetened beverages, dietary risks, and high alcohol use (**Figure 5**). Tamil Nadu also ranks highest in high BMI, low physical activity, and particulate matter pollution, indicating critical lifestyle and environmental health concerns. In contrast, Bihar, Rajasthan, and Jharkhand consistently rank lower (30th and 31st) across categories like diet low in fruits, whole grains, smoking, and secondhand smoke exposure. Nagaland and West Bengal also frequently rank lower in terms of various health risks. Manipur and Uttarakhand rank high (1st and 2nd) in categories such as tobacco use, smoking, and secondhand smoke exposure. Haryana and Arunachal Pradesh rank higher across a range of factors like dietary risks, high BMI, and tobacco-related factors. Andhra Pradesh and Delhi rank in the middle across various factors, showing moderate risks in alcohol use, dietary risks, and physical inactivity.

**Figure 6:**
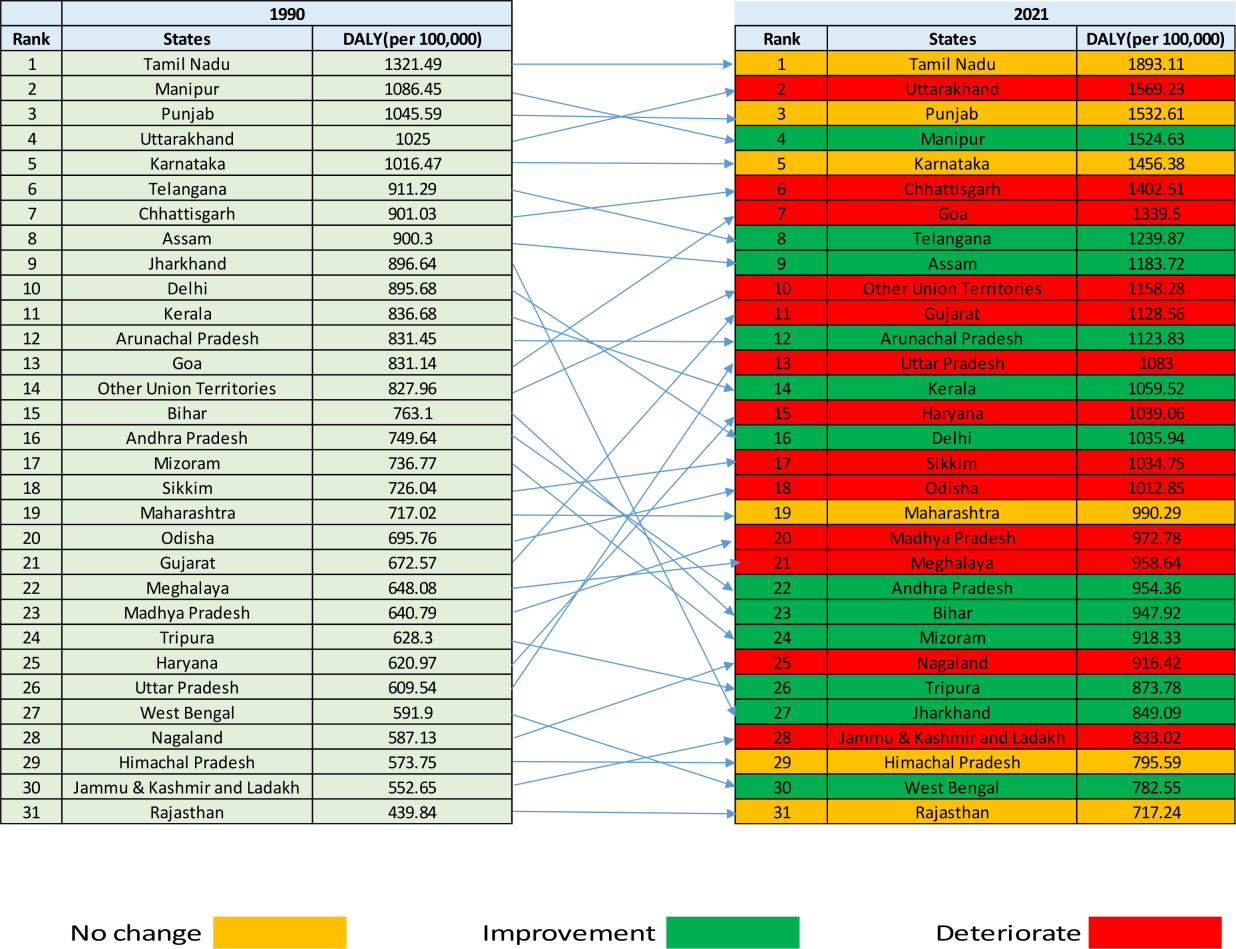
Change in the rank of diabetes in terms of age standardize rate of DALY in India, 1990-2021. DALY, disability-adjusted life-year.

The analysis of DALY between 1990 and 2021 reveals significant shifts in health outcomes across Indian states, which can be categorized into deteriorated, improved, and stable groups. In the deteriorated group, Uttarakhand saw a rise in DALY from 1025 to 1569.23 (ranked 4th to 2nd), Chhattisgarh increased from 901.03 to 1402.51 (7th to 6th), Goa moved from 831.14 to 1339.5 (13th to 7th), and Other Union Territories rose from 827.96 to 1158.28 (14th to 10th) (**Figure 6**). Similarly, Gujarat escalated from 672.57 to 1128.56 (21st to 11th), Uttar Pradesh from 609.54 to 1083 (26th to 13th), Haryana from 620.97 to 1039.06 (25th to 15th), and Sikkim from 726.04 to 1034.75 (18th to 16th), while Odisha, Madhya Pradesh, Meghalaya, Nagaland, and Jammu & Kashmir and Ladakh also exhibited increased DALY values, Conversely, in the improved group, Manipur, though its DALY increased from 1086.45 to 1524.63, dropped from 2nd to 4th, reflecting relative improvement compared to others. Telangana moved from 6th to 8th, Assam from 8th to 9th, and Arunachal Pradesh maintained its rank at 12th, while Kerala improved from 11th to 14th, Delhi shifted from 10th to 16th, and Andhra Pradesh improved from 16th to 21st. Bihar, Mizoram, Tripura, Jharkhand, and West Bengal also registered better health outcomes. Meanwhile, the stable group included Tamil Nadu, which consistently ranked 1st in both 1990 and 2021, with its DALY increasing from 1321.49 to 1893.11, while Punjab remained 3rd, Karnataka held its position at 5th, and Maharashtra stayed at 19th, indicating a persistent health burden. Himachal Pradesh maintained 29th rank, and Rajasthan consistently had the lowest DALY values, ranking 31st in both years.

**Figure 7:**
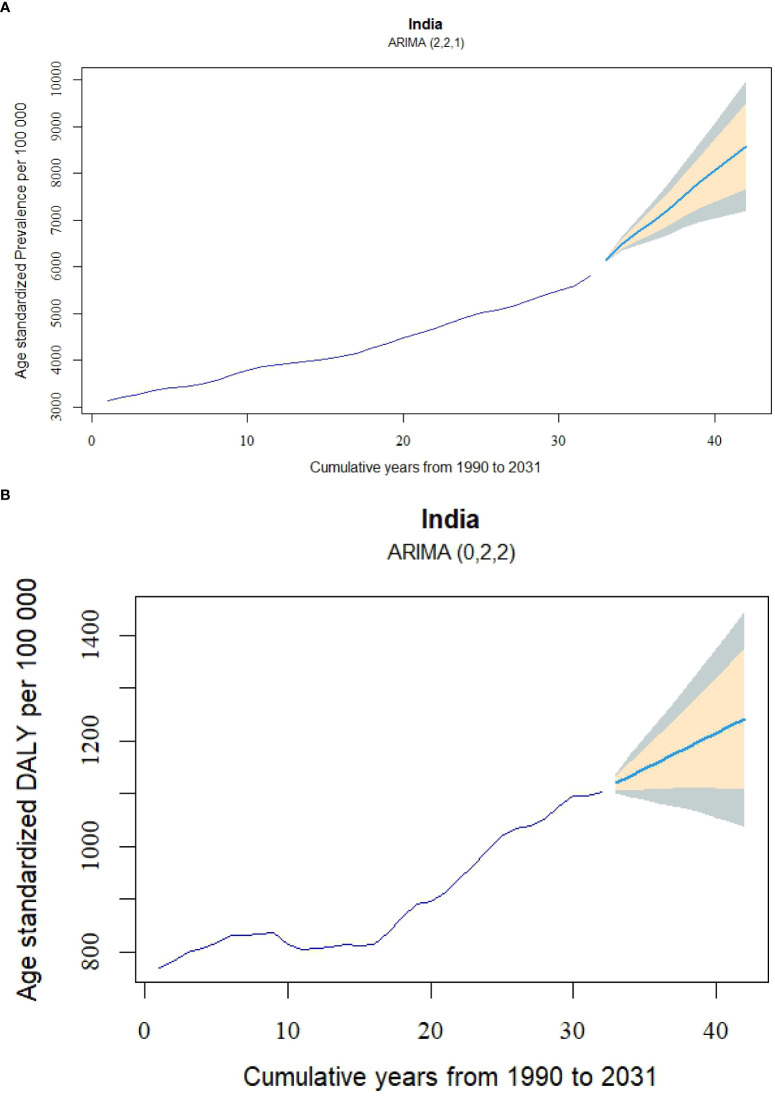
Age-standardized DALY and prevalence rate forecasts for diabetes in India from 1990 to 2031, using ARIMA Models. **(A)** Prevalence rate forecast using the ARIMA (2,2,1) model, and **(B)** DALY rate forecast using the ARIMA (0,2,2) model. DALY, disability-adjusted life-year.

The forecasted age-standardized prevalence rates per 100,000 population in India from 1990 to 2031 reveals a marked upward trend. The prevalence rate is projected to increase steadily from 6150.19 (95% CI: 6109.18 to 6191.21) in 2022, reaching 6960.33 (95% CI: 6549.48 to 7371.18) by 2025, and further rising to 7512.59 (95% CI: 6828.48 to 8196.7) by 2027 (**Figure 7A**). By 2031, the prevalence rate is expected to peak at 8585.45 (95% CI: 7190.57 to 9980.33). This consistent upward trajectory highlights a growing burden of disease over time, as reflected in the forecasted values (**Supplementary Table 2**). Although the projections suggest a marked upward trend, they represent estimates under the assumption that current trends continue.

**Figure 7B** presents a significant upward trend in the age-standardized DALYs per 100,000 population in India, projecting a continuous increase from 2022 to 2031. In 2022, the DALYs rate is forecasted to be 1119.69 (95% CI: 1101.08 to 1138.31), with the upward trajectory persisting over time. By 2025, the DALYs rate is expected to rise to 1160.32 (95% CI: 1081.94 to 1238.7), and by 2031, it is projected to reach 1241.57 (95% CI: 1036.2 to 1446.94). The forecast indicates that the DALYs rate will surpass the 1200 threshold by 2028, where it is estimated at 1200.94 (95% CI: 1062.65 to 1339.23). It also highlights the increasing uncertainty in the projections, as evidenced by the widening confidence intervals approaching 2031, indicating that the actual disease burden may vary within a broader range as time progresses.

## Discussion

The rising incidence of diabetes in India from 1990 to 2021 represents a growing public health challenge. Over these 30 years, the national incidence rate has surged by over 62%, reflecting the rapid spread of diabetes, particularly in Southern and Western states like Tamil Nadu, Goa, and Karnataka. While the national trend underscores an overall increase in diabetes cases, regional disparities paint a more complex picture of the epidemic’s impact across the country. These states, known for their higher urbanization rates and lifestyle changes, have experienced the steepest increases in diabetes incidence and its associated health burdens (22, 23).

In Tamil Nadu, the ASPR for diabetes in 2021 reached 8,299.55 per 100,000, significantly higher than the national average. This represents an increase of nearly 140% since 1990, driven by a combination of dietary risks, sedentary lifestyles, and urbanization (4, 22, 24). Similarly, Goa and Karnataka have experienced comparable increases in ASPR, aligning with existing evidence that indicates a high and growing prevalence of diabetes, hypertension, dyslipidemia, and obesity across India factors that may underlie these rising trends (10). These findings were comparable to the other studies which shows that Obesity and physical inactivity are major lifestyle-related risk factors for the development of Type 2 Diabetes Mellitus (T2DM). Research has demonstrated that modest weight loss, achieved through dietary changes and increased physical activity, can reduce the incidence of T2DM by 40% to 60% in individuals at high risk (25). Furthermore, unhealthy dietary patterns, particularly those characterized by excessive caloric and fat consumption, are prevalent in urbanized and industrialized areas, further exacerbating the diabetes epidemic (26). Type 2 diabetes mellitus (T2DM) is highly prevalent in India, and among these individuals, erectile dysfunction (ED) is frequently observed. Key risk factors for ED in T2DM patients include advanced age and prolonged duration of diabetes (27).

Although the incidence of diabetes has risen dramatically, the increase in mortality has been more modest, growing by 34.8%. The slower rise in disease cases may be attributed to improvements in healthcare systems, particularly in diagnosis and treatment. The Ayushman Bharat Health and Wellness Centre play a key role in the screening and early detection of NCDs, including diabetes. These centers also focus on raising awareness about risk factors, which likely contributes to enhanced disease prevention and management (28). States like Jharkhand exemplify this trend, where the mortality rate has slightly declined despite a steady increase in incidence. In contrast, Uttarakhand and Punjab, which have seen concurrent increases in both incidence and mortality, demonstrate that healthcare systems in some regions are struggling to keep pace with the growing number of cases. Addressing the burden of diabetes in India requires comprehensive public health strategies that focus on prevention, education, and access to healthcare services (4).

The divergence between incidence and mortality in different states points to disparities in healthcare access and disease management, with some states better equipped to handle the growing diabetes burden. The rate ASPR of diabetes has nearly doubled nationally, likely driven by a combination of rising affluence and increased public awareness. While higher affluence has been associated with a growing prevalence of diabetes due to lifestyle changes, heightened awareness of the disease may contribute to its better management and potential mitigation. The ASPR of diabetes almost doubled nationally, increasing by 84.76% in 2021 consistent with other study findings that diabetes prevalence is rising in India and it is driven by a combination of rising affluence and increased public awareness. While higher affluence has been associated with a growing prevalence of diabetes due to lifestyle changes, heightened awareness of the disease may contribute to its better management and potential mitigation (29). Similarly, the DALY rate, a measure of the overall disease burden, rose by 43.34%​ which is in line with findings from other global studies with the observation that the worldwide burden of diabetes has increased significantly since 1990, and forecasts suggest that it will continue to grow by 2025 (30). The sharp rise in diabetes prevalence and disease burden, particularly after 2005, highlights its growing impact on India’s healthcare system, especially in Southern and Western states like Tamil Nadu and Goa, which report the highest ASPR and disease burden. Significant differences in disease burden across states suggest varying levels of diabetes management, with Tamil Nadu having the highest rate in 2021 at 1,893.11 DALY per 100,000, while states like West Bengal and Himachal Pradesh showed relatively lower rates. Similar trends were observed in the work of R Pradeepa and Misra et al. where they attributed this disparity to factors such as urbanization and lifestyle changes, which are more pronounced in Tamil Nadu. These changes may have amplified diabetes risk, leading to a higher disease burden, while states like West Bengal and Himachal Pradesh, with differing socio-economic conditions and lifestyle patterns, have likely seen a relatively lower impact (31, 32).

Age and gender disparities further complicate the national diabetes picture. The disease disproportionately affects older adults, with men showing higher prevalence rates across nearly all age groups. This is corroborated by multiple studies where men in India have a higher prevalence of diabetes compared to women and older adults are more susceptible to diabetes due to factors like decreased physical activity and increased body mass index (32, 33). In 2021, men aged 65–69 exhibited a prevalence of 19,380.75 per 100,000, significantly higher than women in the same age group. This gender gap, which becomes more pronounced with age, is likely influenced by risk factors such as higher rates of smoking, alcohol consumption, and hypertension among men which is in line with the observations of other studies which also indicates that men exhibit higher rates of smoking and alcohol consumption, both of which are critical risk factors that contribute to the development of diabetes and its associated complications. These behaviors are associated with poorer glycemic control and a heightened risk of cardiovascular diseases in men compared to women (34). Additionally, global studies indicates that men experience higher rates of hypertension, further increasing their susceptibility to cardiovascular complications linked to diabetes (35). These findings underscore the importance of gender-sensitive health interventions and the need to target older populations with preventive measures​​. RK Rana et al. observed the prevalence of hypertension (HTN), diabetes mellitus (DM), and their concurrent presence has increased in India, as shown by a comparison of the NFHS-4 and NFHS-5 data sets. The rise in prevalence is particularly noticeable in the age group of 30-50. This indicates the importance of screening for both conditions in patients visiting clinics for either diabetes or heart care, suggesting that integrated screening could enhance early detection and management (36). In the Indian context, where Ayurveda has a long-standing tradition, current evidence supports the potential benefit of various Ayurvedic medicines in improving glycemic control among patients with type 2 diabetes mellitus (T2DM). However, high-quality randomized controlled trials (RCTs) should be conducted and reported to validate their safety, efficacy, and potential integration into standard medical practices (37).

The forecasted trends for diabetes in India indicate a consistent upward trajectory in both the prevalence of the condition and its associated burden from 2022 to 2031. The projected increases in age-standardized prevalence rates and DALYs suggest a growing public health concern. The steady rise in prevalence rates through 2031, alongside the anticipated surpassing of 1200 DALYs per 100,000 population by 2028, highlights the potential for an increased strain on healthcare systems. Furthermore, the widening confidence intervals toward 2031 point to an increasing range of possible outcomes, suggesting variability in the future burden of the disease. They should be interpreted with the understanding that future changes in policy, behavior, or other factors could alter the trajectory. These projections emphasize the importance of timely and targeted public health strategies to manage the evolving impact of diabetes in India. The study’s strength lies in its use of ARIMA models to project long-term trends in diabetes prevalence and burden from 2022 to 2031, based on comprehensive historical data. It provides valuable state-wise analysis, identifying regional disparities and highlighting high-burden areas, while utilizing multiple metrics such as prevalence, DALYs, and mortality for a well-rounded understanding of the diabetes burden in India. There are some limitations. First, the reliance on secondary data from the GBD database may introduce inconsistencies due to varying data collection methods and reporting standards across different regions. Second, GBD estimates are derived from a modeling process that synthesizes multiple data sources of heterogeneous quality, potentially contributing to measurement bias and uncertainty in our findings. Finally, although ARIMA models provide valuable long-term projections, external factors such as healthcare policy changes, unanticipated interventions, and broader socioeconomic shifts could alter future trends beyond what our models capture. Future research can address these limitations by incorporating socioeconomic variables to improve the accuracy of projections and provide deeper insights into regional disparities.

## Conclusion

This study reveals a significant and consistent rise in the prevalence and burden of diabetes in India from 1990 to 2021, with forecasts indicating a continued upward trend through 2031. States such as Tamil Nadu, Goa, and Karnataka exhibit the highest age-standardized prevalence rates and DALYs, identifying them as areas with the most urgent need for targeted interventions. The analysis points to key risk factors driving the diabetes burden, including dietary risks, physical inactivity, and high BMI, which are particularly pronounced in high-burden regions. These findings emphasize the need for region-specific public health strategies that focus on addressing these modifiable risk factors. Strengthening preventive measures and healthcare systems, especially in the most affected states, will be crucial in mitigating the growing impact of diabetes in India. To address the escalating diabetes burden, our findings advocate for a dual prevention strategy. At the primary prevention level, it is imperative to implement community-based initiatives, including comprehensive health education programs, dietary counseling, and structured physical activity interventions. Such measures are essential to mitigate key modifiable risk factors, notably high BMI and sedentary behavior. Concurrently, secondary prevention should prioritize systematic early detection through regular screening and timely clinical intervention for individuals at risk or in the initial stages of diabetes. This combined approach is vital to impede disease progression and diminish the incidence of complications, particularly in regions exhibiting the highest prevalence rates.

## Data Availability

Publicly available datasets were analyzed in this study. This data can be found here: https://vizhub.healthdata.org/gbd-results/.
